# Implementation of cell-free DNA-based non-invasive prenatal testing in a National Health Service Regional Genetics Laboratory

**DOI:** 10.1017/S0016672319000119

**Published:** 2019-12-09

**Authors:** Fiona S. Togneri, Mark D. Kilby, Elizabeth Young, Samantha Court, Denise Williams, Michael J. Griffiths, Stephanie K. Allen

**Affiliations:** 1 West Midlands Regional Genetics Laboratory, Birmingham Women's and Children's NHS Foundation Trust, Edgbaston, B15 2TG, UK; 2 Fetal Medicine Centre, Birmingham Women's and Children's NHS Trust, Edgbaston, B15 2TG, UK; 3 Institute of Metabolism & Systems Research, University of Birmingham, B15 2TT, UK; 4 West Midlands Regional Genetics Service, Birmingham Women's and Children's NHS Foundation Trust, Edgbaston, B15 2TG, UK

**Keywords:** aneuploidy, cfDNA, implementation, NHS, NIPT

## Abstract

**Background:**

Non-invasive prenatal testing (NIPT) for the detection of foetal aneuploidy through analysis of cell-free DNA (cfDNA) in maternal blood is offered routinely by many healthcare providers across the developed world. This testing has recently been recommended for evaluative implementation in the UK National Health Service (NHS) foetal anomaly screening pathway as a contingent screen following an increased risk of trisomy 21, 18 or 13. In preparation for delivering a national service, we have implemented cfDNA-based NIPT in our Regional Genetics Laboratory. Here, we describe our validation and verification processes and initial experiences of the technology prior to rollout of a national screening service.

**Methods:**

Data are presented from more than 1000 patients (215 retrospective and 840 prospective) from ‘high- and low-risk pregnancies’ with outcome data following birth or confirmatory invasive prenatal sampling. NIPT was by the Illumina Verifi® test.

**Results:**

Our data confirm a high-fidelity service with a failure rate of ~0.24% and a high sensitivity and specificity for the detection of foetal trisomy 13, 18 and 21. Secondly, the data show that a significant proportion of patients continue their pregnancies without prenatal invasive testing or intervention after receiving a high-risk cfDNA-based result. A total of 46.5% of patients referred to date were referred for reasons other than high screen risk. Ten percent (76/840 clinical service referrals) of patients were referred with ultrasonographic finding of a foetal structural anomaly, and data analysis indicates high- and low-risk scan indications for NIPT.

**Conclusions:**

NIPT can be successfully implemented into NHS regional genetics laboratories to provide high-quality services. NHS provision of NIPT in patients with high-risk screen results will allow for a reduction of invasive testing and partially improve equality of access to cfDNA-based NIPT in the pregnant population. Patients at low risk for a classic trisomy or with other clinical indications are likely to continue to access cfDNA-based NIPT as a private test.

## 1. Background

Current recommended and UK National Health Service (NHS)-funded antenatal screening for foetal aneuploidies is by combined first-trimester ultrasound and biochemical maternal serum screening. Pregnant patients with a post-test risk of ≥1:150 for trisomy 21, 18 or 13 in England are offered invasive testing (amniocentesis or chorionic villus sampling (CVS)) for definitive genetic diagnosis (1). Increasingly, however, UK patients are accessing cell-free DNA (cfDNA)-based non-invasive prenatal testing (NIPT) either in place of the NHS screening pathway or for clarification of risk prior to accessing invasive procedures following high-risk results.

NIPT analyses cfDNA in the blood plasma of pregnant patients (2). Ten percent of this cfDNA is placental in origin and as such acts a proxy for the foetus (2,3). Quantifying chromosome-specific amounts of foetal cfDNA allows for screening for aneuploidies. Next-generation sequencing is employed by the majority of NIPT technologies to directly measure the genomic representation of each chromosome and to determine when the contribution of chromosomes 13, 18 or 21 is increased in relation to other autosomes, thereby indicating a high chance of the respective trisomy in the foetus (4–7). This testing offers a higher-performance screen than current screening methods, can be used contingently and promises to reduce invasive testing (and therefore risk of foetal loss) in unaffected pregnancies (8–12). In the UK, NIPT is currently offered via locally funded services in some areas or at a cost to patients presenting at private healthcare providers and some NHS providers, with much of this testing taking place within commercial companies. Recently, the UK National Screening Committee (UK NSC) recommended evaluative implementation of cfDNA-based NIPT as a contingent screen for patients with high-screen-risk results (≥1:150), to be performed in NHS pathology laboratories (13).

In anticipation of this UK NSC recommendation, an NIPT service was established at the West Midlands Regional Genetics Laboratory (WMRGL) in Birmingham, UK, in September 2015. Here, we present data on this implementation prior to NHS funding being available for high-screen-risk patients in England. We look at the different groups of patients being referred for this testing and discuss our experience to date and alternative applications of NIPT.

## 2. Methods

### 2.1. Technology selection

An invitation to tender was released in December 2014. Commercial companies were invited to propose an NIPT solution for detection of trisomy 13, 18 and 21, to include reagents, a validated protocol, software and technical transfer with ongoing support. The Illumina Verifi® test was selected at the time due to its simple and easy-to-implement technical workflow, significant published evidence of superior performance statistics (14) and the availability within the laboratory of a HiSeq 2500 capable of performing the assay.

### 2.2. NIPT assay

Plasma was isolated from maternal samples by centrifugation. Total cfDNA was then extracted using QIAmp DNA Blood Mini Kit column-based DNA extraction kits (Thermo Fisher Scientific, USA) according to the manufacturer's instructions. Sequencing libraries were prepared using Illumina's TrueSeq Nano DNA LT Sample Preparation Kit (Illumina, USA) and libraries from 15 plasmas were pooled and loaded onto a HiSeq 2500 next-generation sequencer for injection into each flowcell. Following flowcell cluster generation by bridge amplification, massively parallel whole-genome sequencing was performed across 36 sequencing cycles using Illumina's 50-cycle rapid run kits. Single end reads of 36 bp were obtained over a 7-hour period, producing ~18.75 million reads per sample. An Illumina-provided bioinformatics pipeline called cADAS was then activated for detection of aneuploidy from the sequencing data. Fragments were aligned, duplicates removed and reads counted thereafter.

Key quality metrics were measured throughout the assay following cfDNA extraction, library preparation and sequencing. Samples not meeting criteria prior to sequencing were failed manually. Following sequencing, cluster density of the flowcell, number of indexed reads per sample, ratio of reads per unique genome location, GC bias and likelihood scores for the result were established (among other parameters), and using this information, a quality control (QC) pass, warning or fail was assigned by the algorithm for each sample. A normalised chromosome value (NCV) score was established per sample for 13, 18 and 21 to indicate the likelihood of trisomy (15), comparing the number of reads for these chromosomes against other autosomes. An NCV score of greater than 4 (for chromosomes 13, 18 or 21) was considered significant. The NCV for X was only analysed in the presence of scan features consistent with Turner syndrome and consent for testing. Numbers of reads were routinely studied for all autosomes.

### 2.3. Patient samples

This study is a combined analysis of: (1) two retrospective patient cohorts (population 1 and 2) and (2) a single prospective patient cohort (population 3) of patients electing for NIPT for trisomy 13, 18 and 21 at WMRGL. Maternal plasma was isolated from blood in Streck™ blood collection tubes from patients with singleton pregnancies at >10 weeks’ gestation, <5 days after sampling. Maternal blood samples were taken prior to any invasive procedures being carried out. The majority of patients were reported as being ‘highly likely’ to have a foetus with trisomy 13, 18 or 21 or ‘highly unlikely’. Data were analysed blinded. Two patients from the clinical service (patient population 3) were reported as having an ‘increased chance’ due to low NCV scores in the positive range (NCV scores for these patients were greater than the cut-off of 4, but lower than those observed for other trisomies in the dataset and lower than expected based on foetal fraction). Only results from patient population 3 (clinical service) were reported to patients.

### 2.4. Validation and verification

#### 2.4.1. Patient population 1

Single aliquots of anonymized, non-trisomic patient plasmas were provided by Illumina as part of their technical transfer process.

#### 2.4.2. Patient population 2

Samples were obtained from pregnant patients recruited to three research studies; PAGE (Prenatal Assessment of Genomes and Exomes; Research Ethics Committee reference numbers (RECs): 14/WM/0150 and 14/WM/1219), RAPID (Reliable Accurate Prenatal Non-Invasive Diagnosis; REC: 01/0095) and NIPSIGEN (Non-Invasive Prenatal Diagnosis for Single Gene Disorders; REC: 13/NW/0580).

- PAGE samples were from local pregnant patients undergoing invasive testing due to abnormal scan findings. Plasmas were isolated for NIPT and anonymized and blinded prior to banking.
- RAPID plasmas were obtained from the RAPID biobank in London (16) and were nationally recruited from patients undergoing invasive testing (for any reason); these samples were received blinded with an aneuploidy frequency of ~10%.
- NIPSIGEN samples were from local pregnant patients recruited as control samples to the NIPSIGEN study (17), but excluded from that project due to foetal aneuploidy.

### 2.5. Clinical service

#### 2.5.1. Patient population 3

NIPT was offered, predominantly as a private service, to patients presenting at four NHS maternity units – Birmingham Women's and Children's NHS Trust, The Royal Wolverhampton NHS Trust, The Hillingdon Hospitals NHS Foundation Trust and Imperial College Healthcare NHS Trust – or at the Regional Genetics Service at Birmingham Women's and Children's NHS Trust. Follow-up information was obtained through communication (telephone and e-mail) with clinicians or by audit of the laboratory database looking at amniocentesis, CVS, foetal tissue and postnatal blood samples received by our laboratory following NIPT results.

Prior to implementing clinical services, multiple opportunities were taken for user education. An evening educational event was held in central Birmingham with colleagues from maternity, genetics and foetal medicine invited to learn more about the technology and the proposed service. Local seminars were also given at various West Midlands trusts. A user referral pack was designed, uploaded electronically to the laboratory and Birmingham Women's and Children's NHS Trust maternity directorate websites and issued to all users. The pack includes an information leaflet for referring clinicians, an instruction guide for clinicians on how to refer patients for this testing, a consent form that must be completed and returned to the laboratory at time of request and a patient information leaflet to be used in counselling for this testing. The patient information leaflet was adapted from that produced by the RAPID study in collaboration with local genetics and maternity colleagues. In anticipation of nationally funded NIPT services, the NHS foetal anomaly screening programme has delivered a standardized education and training programme across NHS trusts for all healthcare professionals involved in pre- and post-test counselling.

## 3. Results

### 3.1. Patient population 1

A total of 75 plasmas were processed (Table 1); no follow-up or patient clinical information (other than absence of trisomy 13, 18 or 21) was available. QC results showed success for all but one sample. This sample had a low library concentration. No repeat plasma was available. Results were euploid for 73/74 samples processed. One patient showed apparent monosomy for chromosome 18.

**Table 1.** Clinical metrics across the three cohorts.

Metric	Population 1 (technical validation)	Population 2 (clinical verification)	Population 3 (clinical service)	Combined (full dataset)
Number	75	140	840	1055
Median gestational age (weeks)	–	15	14	14
Number with gestational information	–	123	791	914
First trimester, n (%)	–	49 (40)	391 (49.4)	440 (48)
Second trimester, n (%)	–	69 (56)	383 (48.4)	452 (49.5)
Third trimester, n (%)	–	5 (4)	17 (2.2)	22 (2.5)
Turnaround time (calendar days)	N/A	N/A	8.5	–
Reason for referral(number with information)	–	70	746	816
High risk from ANS^*a*^, n (%)	–	–	396 (53.5)	–
Maternal request, n (%)	–	–	187 (24.5)	–
Previous trisomy, n (%)	–	–	72 (10)	–
Ultrasound scan findings, n (%)	–	70	76 (10)	146
Robertsonian translocation^*b*^, n (%)	–	–	5 (1)	–
Missed screening, n (%)	–	–	10 (1)	–
Cancellations at sample receipt, n (%)	N/A	N/A	3 (0.36)	–
Technical failure at first run, n (%)	1 (1.3)	3 (2.2)	2 (0.24)	6 (0.57)

^*a*^ ≥1 in 150 risk of trisomy from the combined screening test in the first trimester or the quad test in the second trimester.

^*b*^ One parent a known carrier of a balanced Robertsonian translocation.

N/A and ‘–’ indicate data as unavailable or metric not applicable to that dataset.

ANS = Antenatal screening.

### 3.2. Patient population 2

A total of 140 patient plasmas were processed as part of clinical verification studies. Patients had a median gestational age of 15 weeks (range 11–32 weeks) (Table 1). Three samples (3/140, 2%) failed to produce a result at first run; for two of these, a result was obtained from a second aliquot from the same blood sample. One sample had insufficient plasma for re-run and failed to yield a result. Twenty-five percent of samples tested gave positive NIPT results: 9 trisomy 13, 10 trisomy 18 and 16 trisomy 21 (Table 2). No discordant results were observed.

**Table 2.** Autosomal trisomic aneuploidy incidence and performance statistics across the three cohorts.

Result	Population 1	Population 2	Population 3	Combined	Sensitivity (95% CI)	Specificity (95% CI)
Trisomy 21	0	16	31^*a*^	47	100 (90.5–100)	100 (99.5–100)
Discordant, n	–	0	0	0	–	–
Trisomy 18	0^*b*^	10	6	16	100 (75–100)	99.9 (99.4–100)
Discordant, n	–	0	1^*c*^	1	–	–
Trisomy 13	0	9	5	14	100 (69.9–100)	99.8 (99.2–100)
Discordant, n	–	0	2^*d*^	2	–	–
Euploid	74	104	795	973	–	–
Discordant, n	0	0	1^*e*^	1	–	–

^*a*^ An additional two patients were reported with borderline results for trisomy 21, data not included here.

^*b*^ One patient showed evidence of monosomy 18.

^*c*^ Trisomy for 18p was observed in placental villi from this patient.

^*d*^ Both patients additionally had aneuploidy elsewhere in the genome.

^*e*^ One patient was diagnosed following birth as having mosaicism for trisomy 21.

CI = confidence interval.

### 3.3. Patient population 3

A total of 840 patients were referred to our NIPT clinical service between 14 September 2015 and 16 March 2018. Patients had a median gestational age of 14 weeks (range 9.1–37.3 weeks). Reasons for referral included high screen risk (53.5%), low-risk maternal request (24.5%), previous trisomic pregnancy (10%) or abnormal ultrasound scan findings (10%) (Table 1). Patients with foetuses with congenital anomalies by ultrasound were all offered but declined invasive genetic testing prior to NIPT being offered.

Three samples were rejected on receipt due to samples not meeting criteria (clotted or incorrect tube type). Two samples (2/840, 0.24%) failed to produce a result from first aliquot (Table 1). For one of these, a result was obtained from the same blood sample using a second plasma aliquot (initial fail due to poor-quality library preparation). The second failure was for biological reasons: further analysis revealed a trisomy in one of the denominator chromosomes (trisomy 7).

Thirty-one patients were reported with trisomy 21 results; NCV scores ranged from 8.5 to 53.0. Outcome data confirmed trisomy 21 in all pregnancies. Results were confirmed following invasive testing of the pregnancy in 21 patients. The remaining ten patients (32%) chose to continue their pregnancy without invasive sampling. Trisomy 21 was confirmed following birth in eight of these patients and following miscarriage or stillbirth in the remaining two. Two additional patients had NCV scores of between 4 and 5 for chromosome 21. As these scores were much lower than previously identified true positive NCV scores identified during validation studies or the clinical service, these were treated as borderline scores. Results for these patients were reported more cautiously but still recommending follow-up invasive sampling. Both of these patients were at an increased risk for trisomy from combined screening test results. One patient opted for invasive sampling by amniocentesis and this result showed no evidence of trisomy 21 (NCV = 4.04); the other patient declined invasive sampling, and trisomy 21 was confirmed following birth (NCV = 4.99).

Six patients were reported with a trisomy 18 result; NCV scores ranged from 7.0 to 36.0. Four of these patients opted for amniocentesis. Trisomy was confirmed in three of these patients. In the fourth patient, no evidence of trisomy 18 was found (at amniocentesis or in cord blood at delivery). Analysis of placental DNA after birth, however, showed evidence of additional copies of the short arm of chromosome 18. Analysis of parental samples showed no evidence of a balanced rearrangement. Trisomy 18 was confirmed in the remaining two pregnancies following live birth (NIPT having taken place at 18 weeks due to abnormal scan findings) and miscarriage, respectively.

Five patients were reported with a trisomy 13 result; NCV scores ranged from 6.0 to 52.0. In two of these patients, patterns suggestive of aneuploidy for multiple chromosomes were observed: respectively trisomy 13 and trisomy 21, and trisomy 13 and trisomy 20. In the first case, only trisomy 21 was confirmed at amniocentesis. In the second case, neither trisomy was confirmed at amniocentesis. The remaining three pregnancies showed evidence of trisomy 13 only; results in these pregnancies were all confirmed, one by amniocentesis, one following miscarriage and one following stillbirth at 31 weeks’ gestation.

Four patients were investigated for Turner syndrome (monosomy X) due to abnormal scan findings (usually large nuchal translucency ± foetal hydrops) suggestive of this condition. One of these pregnancies was reported as being highly likely to be affected with monosomy X. No follow-up data were available for this pregnancy.

A total of 795 patients were reported as being highly unlikely to be affected with trisomy 13, 18 or 21. One of these patients was diagnosed with mosaicism for trisomy 21 following birth with facial features consistent with Down syndrome (47,XX,+21[25]/46,XX[5]).

A total of 420 patients were referred due to high-screen-risk results from standard antenatal screening. A total of 394 (93.5%) were reported with no evidence of trisomy, 17 with trisomy 21 (4.3%), 3 with trisomy 18 (0.7%) and 3 with trisomy 13 (0.7%). Two patients were reported with borderline NCV scores for trisomy 21 (as discussed above). One result was not able to be reported due to the presence of another autosomal aneuploidy (biological fail as discussed above; 0.3%). There were two discordant results: one with trisomy 13 and one with trisomy 18 by NIPT, both of which showed no evidence of trisomy following invasive sampling. Twenty-three patients referred due to increased screen risk were correctly identified as carrying trisomic foetuses. Screen risk results in these patients ranged from 1 in 2 to 1 in 121. Eight patients (35%) had a screen risk greater than 1 in 10, five had screen risk figures of between 1 in 10 and 1 in 50 and other screen risk figures were greater than 1 in 50.

## 4. Discussion

NIPT for the detection of trisomy 13, 18 or 21 has been offered at WMRGL for >30 months, and prior to that, blinded retrospective populations were studied for clinical validation and verification. More than 1000 patient samples have been analysed to date, with outcome data available (from invasive sampling or following birth) for most patients reported with a high chance of aneuploidy from NIPT and some patients reported with low-chance results. The results presented show a highly sensitive and specific non-invasive prenatal test.

Forty-seven patient samples show results consistent with trisomy 21, with just one discordant result in a patient reported without aneuploidy by NIPT found to have mosaicism (a known limitation of NIPT) for trisomy 21 postnatally, confirming that cfDNA-based NIPT is an extremely reliable screening test for the detection of trisomy 21.

The data show a technical failure rate of 0.57%, which drops to 0.24% in our clinical service population, where more than one aliquot of patient plasma was available. This failure rate is significantly lower than the 3.5% used for the cost–benefit analysis in the UK NSC consultation (18). A low failure rate is important for improving sensitivity, reducing parental anxiety and reducing clinician counselling time (19).

**Figure 1.**
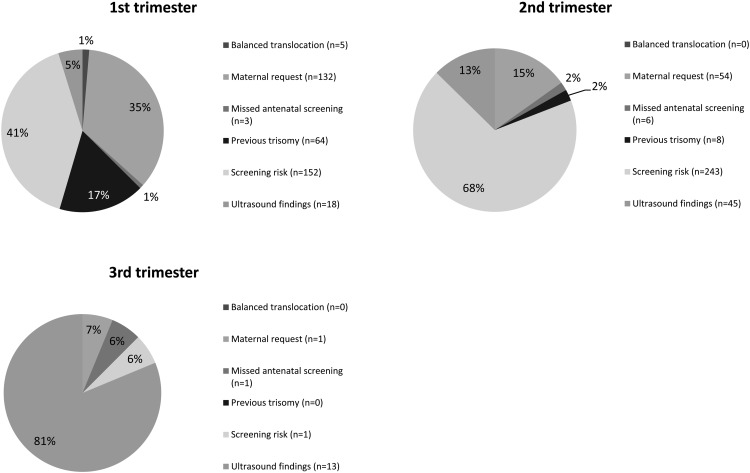
The reasons for patients requesting non-invasive prenatal testing vary according to gestational age (population 3 only). The proportion of patients referred with a high screen risk increases in the second trimester, while patients with previous histories (previous trisomy or translocation carriers) tend to present in the first trimester. The percentage of samples referred due to abnormal scan findings increases with gestational age.

Whilst indications varied according to gestational age (Figure 1), the proportion of patients accessing NIPT as a clinical service was split quite evenly between first and second trimesters. Patients with *a priori* risk from their clinical histories accessed testing early, while those with *a priori* risk from first-trimester combined testing presented later due to the need to await screening results.

**Figure 2.**
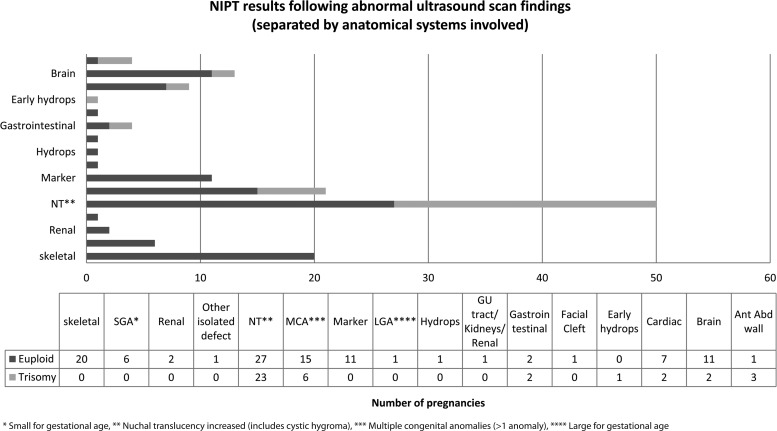
Non-invasive prenatal testing (NIPT) results were available for 146 patients with abnormal scan findings (populations 2 and 3). Nineteen patients had isolated markers only (echogenic bowel, other echogenic foci or short femur) and 127 had foetal structural anomalies, either in a single organ or in multiple organ systems. High-trisomy-risk scan findings include increased nuchal translucency (NT; 46% (23/50) trisomic), multiple congenital abnormalities (MCA; 29% (6/21) trisomic), anterior abdominal (Ant Abd) wall defects (75% (3/4) trisomic) and gastrointestinal defects (duodenal atresia; 50% (2/4) trisomic). Low-trisomy-risk scan findings include echogenic bowel, skeletal defects (including talipes, short long bones and skeletal dysplasia referrals) and babies found to be small for gestational age (SGA). GU = genitourinary.

An interesting cohort of patients (2.2%) requested NIPT in the third trimester. The vast majority of these patients were referred for NIPT analysis based on ultrasound scan findings, with results being used to inform delivery management. We have analysed the scan anomalies present in patients tested by NIPT in both our clinical verification and clinical study cohorts (Figure 2 & Table 3). An aneuploidy result was obtained for almost half of the patients referred due to raised nuchal translucency (3 monosomy X, 2 trisomy 13, 5 trisomy 18 and 13 trisomy 21) and 75% of patients with anterior abdominal wall defects (primarily exomphalos; 1 trisomy 13, 1 trisomy 18 and 1 monosomy X). No patients referred with ultrasound markers on scan only (primarily echogenic bowel or shortened long bones) had NIPT results showing a high chance of aneuploidy.

**Table 3.** Structural defects present at time of referral in patients with positive non-invasive prenatal testing (NIPT) results.

Cohort	Anatomical system	Further clinical details (where available)	NIPT result
2	NT	8.7 mm	Monosomy X
2	NT	Cystic hygroma	Monosomy X
2	MCA	Exomphalos and cystic hygroma	Monosomy X
2	MCA	Echogenic bowel, cardiac foci, AVSD and ventriculomegaly	Trisomy 13
2	NT	3.9 mm	Trisomy 13
2	MCA	–	Trisomy 13
2	NT	6 mm	Trisomy 13
2	Brain	Holoprosencephaly	Trisomy 13
2	Ant Abd wall	–	Trisomy 13
2	MCA	Unilateral choroid plexus cyst, abnormal four-chamber view, clenched hands	Trisomy 18
2	Ant Abd wall	Exomphalos	Trisomy 18
2	Early hydrops	–	Trisomy 18
2	NT	6.7 mm	Trisomy 18
2	MCA	Cystic hygroma, omphalocele and univentricular heart	Trisomy 18
2	NT	Cystic hygroma	Trisomy 18
2	NT	Cystic hygroma	Trisomy 18
2	NT	Cystic hygroma	Trisomy 21
2	NT	Cystic hygroma	Trisomy 21
2	NT	7 mm, possible AVSD	Trisomy 21
2	NT	5.1 mm	Trisomy 21
2	NT	5.7 mm	Trisomy 21
2	NT	9.8 mm	Trisomy 21
2	NT	6.5 mm	Trisomy 21
2	NT	–	Trisomy 21
3	NT	Cystic hygroma	Monosomy X
3	MCA	–	Trisomy 13
3	Cardiac	Multiple muscular VSD	Trisomy 18
3	NT	–	Trisomy 18
3	MCA	AVSD, structural brain abnormalities	Trisomy 18
3	NT	–	Trisomy 21
3	NT	Cystic hygroma	Trisomy 21
3	NT	–	Trisomy 21
3	NT	–	Trisomy 21
3	NT	–	Trisomy 21
3	NT	Cystic hygroma	Trisomy 21
3	Gastrointestinal	Duodenal atresia	Trisomy 21
3	Gastrointestinal	Duodenal atresia	Trisomy 21
3	Brain	Ventriculomegaly	Trisomy 21
3	Cardiac	AVSD	Trisomy 21

Anatomical systems affected in foetuses with structural defects and positive cell-free DNA NIPT results (data represent patients from the PAGE study tested as part of patient population 2, as well as patients from the clinical service (patient population 3)). All patients had foetal structural anomalies. No positive NIPT results were reported in patients with isolated ultrasound markers only. NT = nuchal translucency increased; MCA = multiple congenital anomalies; Ant Abd wall = defects of the anterior abdominal wall; AVSD = atrioventricular septal defect; VSD = ventricular septal defect.

Five percent of patients referred for NIPT due to an increased screen risk from antenatal screening were identified as having aneuploid pregnancies (all confirmed by invasive sampling). Four additional patients underwent invasive sampling following NIPT due to failed, discrepant or borderline NIPT results. Fifty-seven percent of these patients had screen risk figures from standard antenatal screening that were greater than 1 in 50.

Thirty-two percent of pregnancies reported as being highly likely to be affected with Down syndrome as part of our clinical study opted not to undergo invasive testing and continued their pregnancy. This is an interesting observation as there have been ethical concerns that NIPT would lead to increased numbers of terminations in Down syndrome pregnancies and fewer children with Down syndrome (20–24). Our findings are consistent with observations in other publications from the UK (25). Similar observations can be been made in patients with high NIPT-based risks for life-limiting Patau or Edwards syndromes.

We identified four discordant results. In three patients, the NIPT results showed evidence of aneuploidy that was not present following invasive sampling. Such results can be caused by confined placental mosaicism (CPM), undetected twin pregnancies with foetal demise or maternal aneuploidy (e.g., due to neoplasia) (26) or maternal copy-number variants. One such trisomy 18 result from our study was confirmed to be due to CPM. In two patients with discordant trisomy 13 results, an additional aneuploidy was observed. One patient was reported as having no evidence of trisomy by NIPT, and later postnatal testing confirmed mosaicism for trisomy 21. No placental material was available for study. As the foetal cfDNA analysed during NIPT is placental in origin, mosaicism with discrepant populations present in the foetus and placenta can lead to incorrect NIPT results.

Initial NHS funding for the evaluative rollout of NIPT is for patients with high-screen-risk results. The data presented here highlight a group of patients with other clinical indications for NIPT that will not be covered by this clinical pathway. A substantial number of patients are willing to pay for NIPT in place of NHS screening or despite low-screen-risk results. Furthermore, clinicians are requesting NIPT to aid patient management in the absence of consent for invasive sampling. Recent data from Beulen *et al.* (27) highlight that NIPT should not be recommended in place of invasive sampling for genetic evaluation of the aetiology of ultrasound anomalies due in part to limitations in resolution of the analysis (identification of common, whole-chromosome aneuploidy only). The patients presented herein, however, represent a population of patients declining invasive sampling for whom before NIPT provision no genetic results would be available antenatally. In our experience, the introduction of NIPT has been extremely worthwhile for these patient populations, in some instances allowing much improved patient care, such as the actioning of immediate post-birth palliative care options for babies with Patau or Edwards syndromes.

## 5. Conclusions

In conclusion, our experience of implementing NIPT in an NHS Regional Genetics Laboratory has been positive. The technology was straightforward to introduce with appropriate support and materials, and the test has been shown to be reliable, with a failure rate of just 0.24% in clinical practice. The data show patients accessing testing at all stages of pregnancy and with a variety of indications. Widespread NHS provision of NIPT for high-screen-risk patients will provide an improvement to patient care and allow for a reduction in invasive sampling and more equity of testing for some NHS patients; however, it is likely that private NIPT testing will continue alongside this initial NHS implementation. The impact of NIPT provision on pregnancy and delivery management remains to be established. Our data support the notion that a significant proportion of pregnancies are continued without intervention when cfDNA-based NIPT results indicate a high chance for trisomy 21.

## Availability of data and materials

The datasets used and/or analysed during the current study are available from the corresponding author on reasonable request. Most data generated or analysed during this study are included in this published article in the tables provided.
